# Dietary Supplementation of Flaxseed (*Linum usitatissimum* L.) Alters Ovarian Functions of Xylene-Exposed Mice

**DOI:** 10.3390/life12081152

**Published:** 2022-07-29

**Authors:** Radoslava Vlčková, Drahomíra Sopková, Zuzana Andrejčáková, Martina Lecová, Dušan Fabian, Zuzana Šefčíková, Alireza Seidavi, Alexander V. Sirotkin

**Affiliations:** 1 Department of Anatomy, Histology and Physiology, University of Veterinary Medicine and Pharmacy in Košice, Komenského 73, 041 81 Košice, Slovakia; radoslava.vlckova@uvlf.sk (R.V.); drahomira.sopkova@uvlf.sk (D.S.); martina.lecova@student.uvlf.sk (M.L.); 2 Institute of Animal Physiology CBS Slovak Academy of Science, Šoltésovej 4-6, 040 01 Košice, Slovakia; fabian@saske.sk (D.F.); sefcikz@saske.sk (Z.Š.); 3 Department of Animal Science, Rasht Branch, Islamic Azad University, Rasht 41335-3516, Iran; alirezaseidavi@iaurasht.ac.ir; 4 Department of Zoology and Anthropology, Constantine the Philosopher University, Trieda A. Hlinku 1, 949 74 Nitra, Slovakia; asirotkin@ukf.sk

**Keywords:** flaxseed, mouse ovary, steroid hormones, IGF-I, gonadotropins, apoptosis, proliferation, xylene

## Abstract

The aim of the performed study was to examine the ability of xylene, flaxseed, and their combinations to affect morphological and endocrine indexes of murine ovaries. The 72 indexes of secondary and tertiary follicular cells, oocytes, corpora lutea, and ovarian stroma have been quantified: diameter, markers of proliferation PCNA and apoptosis caspase 3, receptors to FSH, oxytocin, estrogen (alpha and beta), and progesterone. In addition, concentrations of the ovarian hormones progesterone, estradiol, and IGF-I in the blood, as well as their production by isolated ovaries cultured with and without gonadotropins (FSH + LH mixture), were determined using histological, immunohistochemical, and immunoassay analyses. The character of xylene and flaxseed effects on ovarian functions in mice depended on the stage of ovarian folliculogenesis. It was shown that flaxseed could mitigate and prevent the major (63%) effects of xylene on the ovary. In addition, the ability of gonadotropins to affect ovarian hormone release and prevent its response to xylene has been shown. The effects of these additives could be mediated by changes in the release and reception of hormones. These observations suggest that flaxseed and possibly gonadotropins could be natural protectors of a female reproductive system against the adverse effects of xylene.

## 1. Introduction

Xylene (dimethyl benzene) is an oil-derived hydrocarbon generated by the chemical industry. It is a liquid volatile organic compound considered one of a number of toxic compounds polluting the environment. It may leak into soil, surface, or groundwater. Because it evaporates very easily, it is assumed that most of the xylene entering the soil or water rises into the air, where it breaks down into less harmful chemicals under the influence of sunlight within a few days. For this reason, xylene rarely occurs in topsoil or surface waters in high concentrations, unless there has been concurrent spillage or long-term contamination [1,2].

High exposure to xylene may be observed in histopathological laboratory technicians especially during the deparaffinization of tissue sections [3]. The organ systems that are most susceptible to xylene are the lungs, skin, central nervous system, liver, and kidneys [4]. Many studies have also shown adverse effects of xylene on human and animal reproduction. It is already well known that long-term exposure to xylene has a negative effect on ovarian activity, especially hormone production and release. The inhalation of para-xylene vapors decreases fecundity in rats, as well as progesterone and estradiol levels in rat blood plasma [5]. Women exposed to aromatic hydrocarbons (e.g., xylene) had reduced levels of luteinizing hormone (LH) [6,7], follicle-stimulating hormone (FSH), and prostaglandin [6], as well as pregnanediol 3-glucuronide (pd3G) and estrone 3-glucuronide (E13G) [7]. In vitro studies showed that xylene was able to affect proliferation, apoptosis, and the release of steroids, peptide hormones, and prostaglandins by cultured murine [8], porcine [9,10,11], and bovine [12] ovarian cells. The adverse effects on the ovary could be due to the ability of xylene to induce oxidative stress and to affect the production and reception of steroid hormones, as well as the intracellular regulators of ovarian cell proliferation and apoptosis [2]. However, the consequences of exposure to xylene on the ovarian state remain to be established. It remains unclear whether in vivo exposure to xylene can induce ovarian folliculogenesis and oogenesis, which in turn could affect the release of ovarian hormones and fecundity. Xylene’s influence on murine reproductive processes has not yet been studied. Therefore, the first aim of the present study was to examine possible structural and endocrine changes in the ovaries of mice after oral xylene intoxication. 

The search for natural and inexpensive regulators of reproductive processes and protectors against the adverse effects of oil-related environmental contaminants (including xylene) is important from both the theoretical and practical perspectives. One such remedy could be the use of functional food—plant products containing flavonoids, which can act as natural adaptogens, antioxidants, and phytoestrogens [2]. Previously, the abilities of some plants (buckwheat, rooibos, *Vitex Agnus cactus* [11]; *Tribulus Terrestris* [12]) and plant molecules (quercetin [9]) to mitigate xylene effects on cultured porcine and bovine ovarian cells have been reported. Nevertheless, the effects of these plant additives have been studied only in the in vitro systems, but these additives were able to prevent only part of the xylene effect. Therefore, the search for reliable and cheap plant protectors could still be achieved.

A previous study has suggested that flaxseed could be one such protector (*Linum usitatissimum*, L.). Its abilities to promote puberty, ovarian growth, folliculogenesis, and oogenesis and affect ovarian steroidogenesis in various species have been shown. These effects could be due to the ability of flaxseed and its constituents to reduce oxidative stress and affect ovarian proliferation and apoptosis, estrogen production, reception, and metabolism. In addition, flaxseed oil can affect reproduction by improving the general metabolic state [2]. There are, however, few reports concerning flaxseed’s influence on murine female reproduction: in this species, dietary flaxseed reduced the number of healthy follicles and increased the number of atretic follicles, as well as plasma estradiol levels [13], ovary size, and steroidogenesis [14]. Dietary flaxseed prevented the xylene-induced apoptosis and degenerative changes in several murine organs [15], but the protective abilities of flaxseed against the reproductive toxicity of xylene have not yet been studied. Therefore, the second aim of the performed study was to examine the ability of flaxseed to affect morphological and endocrine indexes of murine ovaries, as well as to modify the influence of xylene on these indexes. The following indexes in secondary and tertiary follicular cells, oocytes, *corpora lutea*, and ovarian stroma were quantified: diameter; marker and promoter of proliferation/cell cycle PCNA; marker and promoter of cytoplasmic apoptosis caspase 3; and receptors to follicle-stimulating hormone (FSH), to oxytocin, to estrogen (alpha and beta), and to progesterone. In addition, concentrations of ovarian hormones progesterone, estradiol, and insulin-like growth factor I (IGF-I) in blood, as well as their production by isolated ovaries cultured with and without gonadotropin (FSH and LH mixture), were determined. These hormonal indexes are considered markers and regulators of ovarian functions [16,17,18,19]. Proliferating cell nuclear antigen can be a marker and promoter of both ovarian cell proliferation [20] and oocyte maturation [21,22]. Caspase 3 can be both a marker and promoter of autophagy and apoptosis in ovarian [16] and non-ovarian [23] cells and in oocytes [24,25].

## 2. Materials and Methods

### 2.1. Animals, Diet, and Supplements

All experiments were carried out under the approval of the State Veterinary and Food Administration of the Slovak Republic (Approval No. 598/18-221/3) in accordance with EU regulations concerning animal experiments. Experiments were performed under standard conditions in the accredited experimental Institute of Animal Physiology of the Slovak Academy of Science in Košice, Slovak Republic. Female mice of the CD-1 ICR line (Velaz s.r.o., Prague, Czech Republic) aged 35 days were used in this study (at the age of 28 days, they were transported from the breeding facility to quarantine at the experimental facility). Mice were kept at a constant temperature between 20 and 24 °C, with a relative humidity of 45–65%, under a 12:12 photoperiod regimen. Standard plastic boxes were lined with bedding intended for barrier breeding (Lignocel 3-4S; VELAZ s.r.o.). The mice were divided into four groups (20 animals per group): (1) control (no treatment), (2) administration of xylene (10 μL/day), (3) administration of flaxseed (10% of food mixture), and (4) administration of xylene (10 μL/day) and flaxseed (10% of food mixture).

Mice of all groups were fed with crushed pellets (M3; BONAGRO a.s., Blažovice, Czech Republic) at a dose of 4 g/head/day (in two separate doses, at 8:00 a.m. and 3 p.m.). The complete diet for mice and rats contained wheat, soybean, lupine extracted scrap, yeast, wheat bran, dried whey, and rape oil (analytical and nutritive components are shown in Table 1). Water was administered ad libitum. Mice of the xylene-treated groups were exposed to mixed xylene p.a. (Sigma, St. Louis, MO, USA) diluted with water 1:10 and administered each day for 14 days at a dose of 10 µL/head/day (equal to 400 mg/kg/day) per os using a cannula for laboratory animals. The diet of flaxseed-treated groups consisted of the basal diet supplemented with flaxseed (variety Libra; 57% α-linolenic acid, ALA; Dom krmív s.r.o., Spišské Vlachy, Slovak Republic), crushed and mixed with M3 as a 10% concentration substitution for 14 days.

The fatty acid profile of the flaxseed variety used, Libra, is stated in Table 2. For these purposes, we used the same gas chromatography (Shimadzu GC 17, Kyoto, Japan with a flame ionization detector; the measurements were expressed in mol%) method as in our previous research [14].

Estrus in females of each group was induced by male pheromones—bypassing the urine-contaminated male litter (so-called Whitten effect). After about three days, females were transported to male cages for mating. For the next four days, females without copulatory plugs in each group were taken from the males and euthanized by dislocation of the cervical vertebrae. 

### 2.2. Blood Collection, Necropsy, and Morphometric Analysis

Immediately before sacrifice, blood was taken from the eye canal, and the ovaries were collected for further analyses. One ovary/animal from each group was used for histological analysis and the other for preparing ovary culture for in vitro experiments. 

For the routine histology and immunohistochemistry, the ovaries were fixed in 4% paraformaldehyde for 24 h, dehydrated, embedded in paraplast, and cut into 5 µm thick sections using the Leica RM2255 microtome (Leica Microsystems Nussloch GmbH, Nussloch, Germany). Histological sections were stained with hematoxylin and eosin (H-E; AppliChem GmbH, Darmstadt, Germany) and mounted in DPX (Distyrene Plasticiser and Xylene; Buchs, Switzerland). For measuring, the light microscope Eclipse E200 (Nikon, Japan) equipped with the color 15Mpix camera ProgRes^®^ CT3 (Jenoptik, Jena, Germany) was used. Morphometric parameters were evaluated using NIS Elements Br (Nikon). Sizes of ovaries, average diameters of follicles, and corpora lutea (CL) were measured in μm. The follicles were identified according to Griffin et al. [26] so that the primary follicles consisted of oocytes surrounded by a single layer of cuboidal granulosa cells (diameters 50–100 μm), the secondary follicles consisted of oocytes surrounded by a double or stratified layer of granulosa cells without formation of an antral cavity (diameter 100–180 μm), and tertiary follicles were follicles with multi-layered cells containing the antral cavity (diameter 180–300 μm). A minimum of 10 follicles of each category were selected for the measure. Diameters of all ovaries, follicles, and CL were calculated from two perpendicular measurements.

### 2.3. Ovary Culture Preparation and Processing

Immediately after their removal from the animals of either control or experimental groups, ovaries were washed several times in sterile phosphate-buffered saline (PBS, Sigma). Whole ovaries were placed into sterile 24-well plates (Beckton Dikinson; 1 ovary/animal per well) with a sterile culture medium (DMEM/F12 1:1, BioWhittakerTM, Verviers, Belgium; 1 mL) supplemented with 10% FBS (fetal bovine serum, BioWhittakerTM) and 1% antibiotic–antimycotic solution (Sigma) and incubated at 37.5 °C and 5% CO2 in humidified air for 24 h. Half of the ovaries were cultured without additions and half with gonadotropins (Pluset, Barcelona, Spain). Active substances of this preparation represented 50 IU/mL pFSH and 50 IU/mL pLH. Gonadotropins were dissolved in the culture medium and added to the culture wells at a dose of 10 µL/mL/well (equivalent to 0.5 IU FSH and 0.5 IU LH per ml of medium) immediately before culture. 

After 24 h incubation, media from the well plates were aspirated and frozen at −18 °C to await immunoassays.

### 2.4. Immunoassay

Progesterone (P4), estradiol-17β (E2), and insulin-like growth factor I (IGF-I) concentrations were analyzed in 25–100 µL of either blood serum or culture medium. Radioimmunoassay was used for P4 and E2 assessment (ng/mL and pg/mL, respectively) and immunoradiometric assay for IGF-I assessment (ng/mL) according to the manufacturer’s instructions in duplicate as described in our previous studies [27,28]. Values in the culture medium are expressed in ng (P4, IGF-I) or pg (E2) per mg tissue per day.

### 2.5. Immunohistochemical Analysis

Paraffin sections of the ovaries were deparaffinized and rehydrated. For detection, antigen retrieval was performed by boiling the slides in EDTA (pH 9.0; OXTr antibody) or in 10 mm citrate buffer (pH 6.0; the rest of the markers) for 6 min. To block endogenous peroxidase activity, the slides were incubated in TBS (0.05 M Tris-HCl plus 0.15 M NaCl, pH 7.6) with 1% H_2_O_2_ addition for 10 min. To block non-specific binding, the sections were incubated for half an hour with 1% bovine serum albumin. In addition, monoclonal mouse anti-PCNA (dilution 1:250; Santa Cruz Biotechnology Inc., Dallas, TX, USA), anti-caspase 3 (dilution 1:200; Abcam plc., Cambridge, UK), anti-FSHr (dilution 1:250; ThermoFisher Scientific, Waltham, MA, USA), anti-OXTr (dilution 1:200; Abcam), anti-human ERα (dilution 1:250, Dako, Glostrup, Denmark), anti-human ERβ (dilution 1:250, Dako, Glostrup, Denmark), and anti-PRB (dilution 1:250; ThermoFisher) antibodies were applied and incubated overnight at 4 °C. After rinsing with TBST (TBS containing 0.1% Tween20), the sections were incubated with goat anti-mouse secondary antibodies (Dako REAL™ EnVision™/HRP, Rabbit/Mouse (ENV), ready-to-use, Dako) for 2 h. Following the incubation, the specimens were rinsed in TBST followed by TBS. A color reaction was visualized by diaminobenzidine as a chromogen (Dako REAL™ DAB + Chromogen, Dako). The stained sections were counterstained with hematoxylin, rinsed in distilled water, dehydrated, and mounted in DPX. In preparing a negative control for each sample, the primary antibody was omitted. Photographic documentation was obtained by using the same light microscope, camera, and software for image analysis as mentioned earlier (see Section 2.2). To quantitatively evaluate the intensity of the IHC reaction, approximately six images from sections of each examined animal (*n* = 6 for each group) were analyzed by using public domain ImageJ software (National Institutes of Health, Bethesda, MD, USA). The outlines of all cells, which showed immunopositive signals in the ovaries (described previously by Andrejčáková et al., 2021; Vlčková et al., 2022), were marked manually, and then the grey level of marked areas was measured. The intensity of the IHC reaction was expressed as the relative optical density (ROD) of the DAB brown reaction products and calculated using the formula described by Smolen [29]. The positive DAB brown reaction was compared to the negative control in all evaluated parameters. 

### 2.6. Statistical Analyses

The in vivo experiment was performed on 20 animals per group. In groups treated with xylene alone and xylene together with flaxseed, 5 mice per group died. For the analyses, only plug-females were used (C, *n* = 14; X, *n* = 9; F, *n* = 14; XF, *n* = 9). Each in vitro experimental group was represented by 6 culture wells containing one ovary. Each series of experiments was performed twice. The data shown are the means of values obtained in these two separate experiments performed on separate days with separate groups of ovaries. Significant differences between the experiments were evaluated using a one-way ANOVA test using Sigma Plot 11.0 statistical software (Systat Software, GmbH, Erkrath, Germany). The effect of additives was analyzed using a two-way ANOVA with Tukey’s post hoc analysis. Differences from controls at *p* < 0.05 were considered significant and are marked with superscript letters.

## 3. Results

### 3.1. Ability of Xylene to Affect Ovarian State

The oral administration of xylene substantially increased the mortality of mice: at the end of experiments, the mortality of control animals was 0%, but animals treated with either xylene alone or xylene together with flaxseed showed a mortality rate of 25% (5 animals out of 20 died in each group). 

The administration of xylene affected morphometric indexes of some mice ovarian structures: it reduced the diameter of primordial and primary ovarian follicles but increased the diameter of secondary (but not tertiary) follicles. The diameter of oocytes or *corpora lutea* was not influenced by xylene (Table 3). 

**Figure 1:**
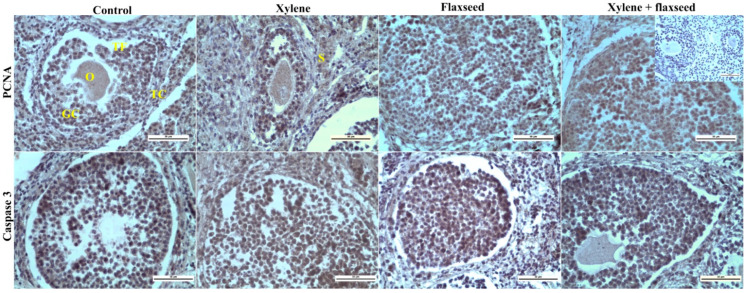
Representative samples of the immunohistochemical reaction of proliferation (PCNA) and apoptotic (caspase 3) markers in the ovaries of mice treated with flaxseed, xylene, and their combinations. PCNA—proliferating cell nuclear antigen; TF—tertiary follicle; GC—granulosa cells; TC—theca cells; O—oocyte; S—stroma. The positive immune reaction (brown) was visualized with diaminobenzidine chromogen (DAB+). Scale bar = 50 µm; magnification 400×. The incorporated picture at a magnification of 400× is an example of a negative control (primary antibodies omitted).

Immunohistochemical analysis of primary follicles could not be statistically evaluated; therefore, only secondary and tertiary follicles were taken into account. Treatment with xylene increased the expression of proliferation marker PCNA in both granulosa and theca layers of secondary ovarian follicles and *corpora lutea* but decreased it in the granulosa (but not in the theca) of tertiary follicles. The PCNA in ovarian stroma did not change after xylene administration. Moreover, treatment with xylene increased the expression of apoptosis marker caspase 3 in the oocytes, granulosa, and theca of secondary follicles, in the granulosa (but not in the oocytes or theca) of tertiary follicles, and in the ovarian stroma, but not in the corpora lutea (Table 4, Figure 1).

**Figure 2:**
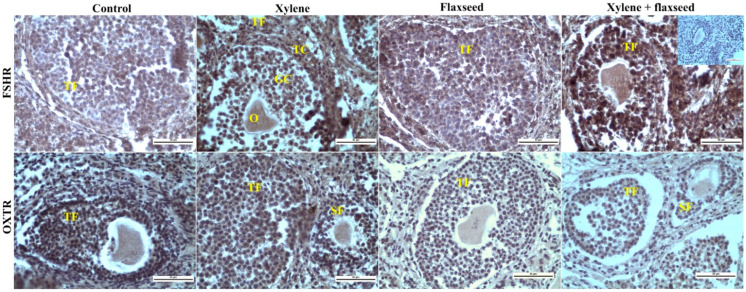
Representative samples of the immunohistochemical reaction of FSHR and OXTR markers in the ovaries of mice treated with flaxseed, xylene, and their combinations. FSHR—follicle-stimulating hormone receptor; OXTR—oxytocin receptor; TF—tertiary follicle; GC—granulosa cells; TC—theca cells; O—oocyte; S—stroma; SF—secondary follicle. Positive immune reaction (brown) was visualized with diaminobenzidine chromogen (DAB+). Scale bar = 50 µm; magnification 400×. Incorporated picture at magnification 400× is an example of a negative control (primary antibodies omitted).

**Figure 3:**
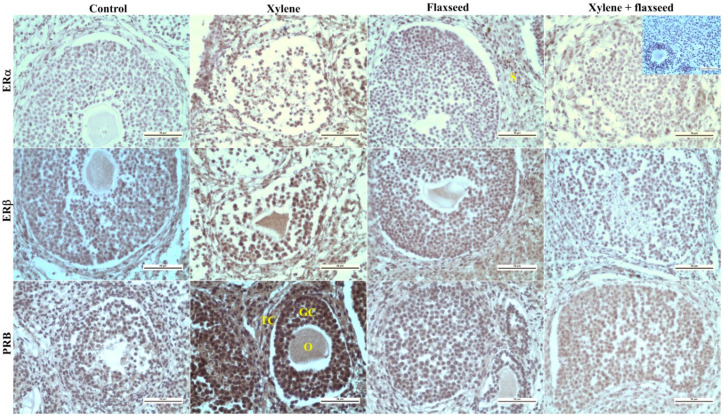
Representative samples of immunohistochemical reaction of ERα, ERß, and PRB in the ovaries of mice treated with flaxseed, xylene, and their combinations. ER—estradiol receptor; PR—progesterone receptor; TF—tertiary follicle; GC—granulosa cells; TC—theca cells; O—oocyte; S—stroma. Positive immune reaction (brown) was visualized with diaminobenzidine chromogen (DAB+). Scale bar = 50 µm; magnification 400×. Incorporated picture at a magnification of 400× is an example of a negative control (primary antibodies omitted).

Xylene was able to affect peptide and steroid hormone receptors (Table 5 and Table 6). The administration of xylene increased the expression of receptors to FSH in oocytes located in both secondary and tertiary follicles, in the ovarian stroma, but decreased it in both the granulosa and the theca of tertiary follicles, but not in the corpora lutea. Xylene also promoted the expression of oxytocin receptors in the oocytes and in the theca of secondary follicles but decreased this expression in the granulosa, theca of tertiary follicles, and corpora lutea (Table 5, Figure 2). We also observed an increase in estrogen receptors α in both the granulosa and the theca of both secondary and tertiary follicles, corpora lutea, and stroma, as well as in oocytes located in secondary follicles. Xylene also increased the expression of estrogen receptor β and progesterone receptors in all the analyzed ovarian structures (Table 6, Figure 3).

**Figure 4:**
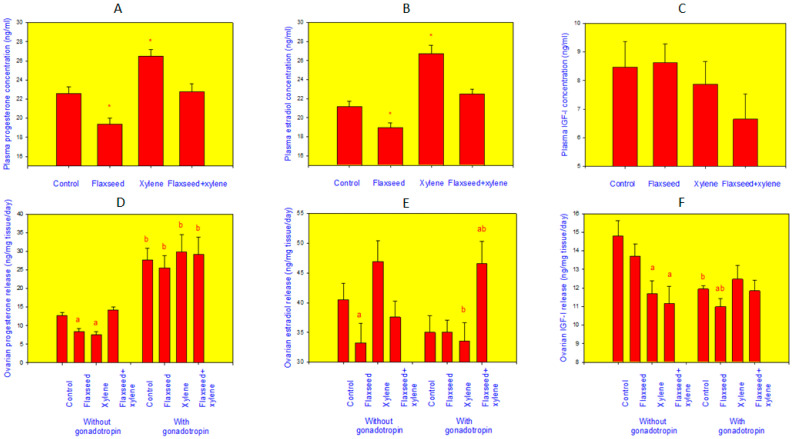
Blood plasma level of progesterone (**A**), estradiol (**B**), and insulin-like growth factor I (IGF-I, (**C**) in mice treated with flaxseed, xylene, and their combinations and the release of progesterone (**D**), estradiol (**E**), and IGF-I (**F**) by the ovaries isolated from mice treated with flaxseed, xylene, and their combinations and cultured with and without gonadotropins (equivalent to 0.5 IU FSH and 0.5 IU LH per mL). The values are mean ± SEM. *—significant (*p* < 0.05) differences with control; a—effect of xylene or flaxseed administration: significant (*p* < 0.05) differences with control; b—effect of gonadotropin: significant (*p* < 0.05) differences between the corresponding groups cultured with and without gonadotropin.

Xylene administration also affected ovarian hormone release. Mice treated with xylene had increased levels of progesterone and estradiol but not of IGF-I in their blood plasma. On the other hand, the ovaries isolated from these mice and cultured without gonadotropin secreted less progesterone and IGF-I than the ovaries of control animals (Figure 4).

### 3.2. Ability of Gonadotropin to Affect Ovarian Functions and to Prevent Xylene Action 

The culture of mice ovaries with gonadotropin increased the release of progesterone and reduced the release of IGF-I, not affecting estradiol output. Moreover, xylene, which reduced progesterone and IGF-I release in the absence of gonadotropin (see above), in presence of gonadotropin failed to affect their release by the cultured ovaries (Figure 4).

### 3.3. Ability of Flaxseed to Affect Ovarian State

In contrast to xylene, the administration of flaxseed did not affect mortality in mice: the number of dead animals at the end of experiments was identical in both the control and the flaxseed-treated group (none of 20, 0%).

The dietary flaxseed reduced the diameter of ovaries, primary follicles, and oocytes in tertiary follicles, but increased the diameter of secondary follicles. Other structures were not affected by flaxseed administration (Table 3). Flaxseed promoted the expression of a marker of proliferation, PCNA, in all the components of secondary follicles and in the corpora lutea, but decreased it in granulosa and theca of tertiary follicles. The expression of apoptosis marker, caspase 3, was increased in oocyte and theca of tertiary (but not secondary) follicles and in the stroma of ovaries in mice fed with flaxseed (Table 4). 

Furthermore, flaxseed administration promoted the expression of FSH receptors in oocytes located in both secondary and tertiary follicles, in the theca of tertiary follicles, and in the ovarian stroma. In other structures, FSH receptors did not change under the influence of flaxseed additive. On the other hand, this additive reduced the expression of oxytocin receptors in all the ovarian structures (Table 5). Flaxseed promoted the expression of estrogen receptors α in all the structures of the tertiary follicle, corpora lutea, and stroma, but not in the secondary follicle. Moreover, administration of flaxseed also increased the expression of estrogen receptor β in all the ovarian structures but decreased the expression of progesterone receptors in the granulosa and the theca (but not in oocytes) in both kinds of follicles and corpus luteum (Table 6). 

Feeding of mice with flaxseed reduced the levels of both progesterone and estradiol, but not IGF-I, in their blood plasma, as well as the release of these hormones by their isolated ovaries (Figure 4).

### 3.4. Ability of Gonadotropin to Prevent Flaxseed Action

The presence of gonadotropin in the culture medium prevented the inhibitory action of flaxseed for progesterone and estradiol release by cultured mice ovaries. On the other hand, the presence of gonadotropin induced the inhibitory action of flaxseed on IGF-I release by these ovaries (Figure 4).

### 3.5. Ability of Flaxseed to Prevent Xylene Action

The mortality rate in animals treated with xylene was increased (up to 25% versus 0% in control; see above). Feeding with flaxseed to animals treated with xylene did not change the mortality rate (it remained 25%).

Flaxseed prevented the action of xylene on the diameter of ovaries and oocytes in secondary follicles, but it did not modify the influence of hydrocarbon on primordial and primary follicles. Moreover, it promoted the action of xylene on the diameter of the secondary follicles and of oocytes in the tertiary follicles (Table 3).

Feeding with flaxseed prevented the action of xylene on cell proliferation (PCNA expression) in all the structures of secondary follicles and in corpus luteum cells. On the other hand, flaxseed promoted xylene’s influence on the proliferation of granulosa in tertiary follicles. Moreover, the combination of xylene with flaxseed reduced the proliferation of stromal cells, although xylene and flaxseed given alone did not affect this parameter. Flaxseed was able to prevent the xylene action on apoptosis (expression of caspase 3) in oocytes of the secondary follicle, in the theca of the tertiary follicle, and in the ovarian stroma, to induce the pro-apoptotic effect of xylene on corpus luteum, but it did not modify xylene action on apoptosis in other ovarian structures (Table 4).

The analysis of peptide hormone receptors showed that flaxseed prevented xylene action on FSH receptors in tertiary follicles (oocytes, granulosa, and theca) and ovarian stroma but not to oocytes in secondary follicles. Furthermore, flaxseed prevented xylene action on oxytocin receptors in oocytes and theca in secondary ovarian follicles but promoted xylene’s influence on oxytocin receptors in all the structures of tertiary follicles, oocytes, and ovarian stromal cells (Table 5).

The analysis of receptors to steroid hormones showed that flaxseed was not able to modify xylene action on estrogen receptors α in any ovarian structure, but it prevented or mitigated xylene action on estrogen receptor β in oocytes and theca in secondary follicles, theca in tertiary follicles, corpus luteum, and stroma. Moreover, flaxseed prevented xylene action on progesterone receptors in all the analyzed ovarian structures (Table 6). 

The analysis of hormones in the blood indicated the ability of flaxseed to prevent xylene action on plasma progesterone and estradiol (Figure 4). Moreover, analysis of hormone release by isolated ovaries showed the ability of flaxseed to prevent xylene action on progesterone but not on IGF-I output. In the presence of gonadotropins, xylene did not affect steroid hormone release, but the ability of flaxseed to prevent xylene action on IGF-I was also retained in the presence of gonadotropins (Figure 4).

## 4. Discussion

The present observations confirm the previous data concerning the presence of markers and regulators of proliferation and apoptosis; receptors to FSH, oxytocin, estrogen, and progesterone; and the ability of ovaries to produce steroid hormones and IGF-I [16,17,18,19]. Moreover, this is the first demonstration of gonadotropin receptors in oocytes presented in mice, which updates the current hypothesis that oocytes do not have gonadotropin receptors and that gonadotropins can regulate oogenesis only through surrounding follicular cells [18].

### 4.1. Ability of Xylene to Affect Ovarian State

In our experiments, the administration of xylene substantially induced mortality in mice. This fact indicates the general and reproductive toxicity of this oil-related environmental contaminant. The living animals treated with this hydrocarbon have smaller primordial and primary follicles and larger secondary but not tertiary ovarian follicles, corpora lutea, or oocytes. These changes were associated with increases in both proliferation (expression of PCNA) and apoptosis (expression of caspase 3) in cells in both granulosa and theca layers of secondary follicles. On the other hand, xylene reduced proliferation and increased apoptosis in granulosa, but not in theca cells of tertiary follicles. These changes in the proliferation:apoptosis rate in granulosa cells did not result in changes in the size of tertiary follicles, which is defined by theca surrounding the granulosa cell layer. These observations demonstrate the ability of xylene to affect mice’s ovarian folliculogenesis, whereas the character and mechanisms of xylene effects depend on the stage of folliculogenesis. Xylene could promote the growth of secondary ovarian follicles via the stimulation of follicular cell turnover but suppress the functions (to inhibit proliferation and to promote apoptosis by granulosa cells) of tertiary follicles.

Xylene was able to influence the size, markers of proliferation, and apoptosis of ovarian structures other than ovarian follicles. It reduced the growth of oocytes in tertiary follicles, which can be explained by increased apoptosis, but not by cell cycle/PCNA. Furthermore, xylene promoted the proliferation (but not apoptosis) of oocytes, which, however, did not influence luteal growth (in terms of the diameter of the corpus luteum). Moreover, xylene promoted apoptosis but not the proliferation of ovarian stromal cells. These observations demonstrate the suppressive action of xylene in an advanced stage of ovarian folliculogenesis, oogenesis, and ovarian stromal cells due to the promotion of apoptotic processes in these structures. On the other hand, the ability of xylene to promote ovarian cell proliferation can explain its ability to be cancerogenic and to induce ovarian cancer [2]. 

These effects of xylene could be induced and mediated by changes in the reception and release of ovarian hormonal regulators. FSH is considered a promoter of ovarian cell proliferation, an inhibitor of their apoptosis, and a promoter of ovarian folliculogenesis and oogenesis [16,19]. Therefore, some xylene-induced changes in these processes could be explained by their influence on FSH receptors. For example, the xylene-induced suppression of functions of tertiary follicles could be the suppression of FSH receptors in these follicles. On the other hand, a xylene-induced increase in FSH receptors in oocytes was associated not with an increase in expression of their PCNA, but with apoptosis/caspase3. This fact indicates that xylene action on oocytes cannot be mediated by FSH receptors. Not only FSH but also oxytocin, IGF-I, and steroid hormones are involved in the control of ovarian functions [16,19] and in mediating oil-related environmental contaminants on these functions [2]. In our experiments, xylene affected the release of steroid hormones and IGF-I, as well as the expression of oxytocin and steroid hormone receptors. All these facts indicate the involvement of oxytocin, IGF-I, steroid hormones, and their receptors in mediating the xylene effect on mice’s female reproductive processes. Nevertheless, understanding the role of these signaling substances in mediating xylene action on mice ovaries requires further examination.

### 4.2. Ability of Flaxseed to Affect Ovarian State

In our experiments, dietary flaxseed did not influence animal mortality, indicating a lack of toxic effects of this plant. Moreover, the present observations demonstrate flaxseed’s effect on ovarian functions. The character of this effect, like the effect of xylene, depended on the stage of ovarian folliculogenesis. It promoted the growth and proliferation of cells in secondary follicles and in corpora lutea, but decreased proliferation and promoted apoptosis in tertiary follicles and their oocytes. These observations suggest that flaxseed can be a stimulator of folliculogenesis and oogenesis in secondary follicles and an inhibitor of these processes in tertiary ones.

The character of flaxseed’s influence on estrogen receptors α is also dependent on the stage of folliculogenesis—plant consumption prompted an expression of these receptors only in tertiary follicles, corpus luteum, and stroma but not in secondary follicles. These facts suggest that some effects of flaxseed on ovarian follicles depend on the stage of folliculogenesis and that these effects could be mediated by estrogens. It is at least known that estrogens and their receptors are generated and play an important role in the promotion of the development of follicles at the late stages of their development [17,18].

On the other hand, the similar action of flaxseed on receptors to FSH, oxytocin, progesterone, and estrogen receptor β in both secondary and tertiary follicles suggest the similar role and mechanisms of action of this plant on antral follicles at all stages of their development. 

The ability of flaxseed to reduce ovarian progesterone and estradiol release both in vivo and in vitro confirms the hypothesis that this plant can affect ovarian functions via changes in the steroid–steroid hormone receptors axis. 

The constituents of flaxseed responsible for its effect require further identification. Nevertheless, the analysis of flaxseed constituents and the similarity of their effects with the effect of the whole plant indicates that flaxseed’s effects on the ovarian cells could be due to the presence of secoisolariciresinol diglycoside, alpha-linoleic acid, and their metabolites—enterolignan enterolactone, docosahexaenoic acid, and 2-methoxyestradiol—as well as antioxidants and phytoestrogens, which are able to affect steroid hormones reception and release [2,14].

The present observations confirm and expand the previous knowledge [2,14] concerning the ability of flaxseed to affect (mainly stimulate) female reproductive processes. They suggest that the character of flaxseed action on ovarian structures can depend on the stage of their development and that this action could be mediated by some peptide and steroid hormones and their receptors. The present studies did not examine the character of flaxseed action on fecundity because they were focused on detecting the mechanisms of action of this plant on ovarian functions. Nevertheless, the results of these studies, together with the previous reports on the stimulatory influence of this plant on animal puberty, ovarian growth, folliculogenesis, oogenesis, and steroidogenesis in various species [2], suggest that this plant could be applicable for the stimulation of reproductive processes as a functional food. Furthermore, it is not to be excluded that flaxseed may be used as a natural adaptogen against the toxic influence of environmental contaminants.

### 4.3. Ability of Flaxseed to Prevent Xylene Action

The following effects of xylene were mitigated, prevented, or even inverted by flaxseed: diameter of ovaries; diameter of oocytes in secondary follicles; PCNA expression in the oocyte, granulosa, and theca of secondary follicles; PCNA expression in *corpus luteum*, expression of caspase 3 in oocyte of the secondary follicle; expression of caspase 3 in the theca and ovarian stroma of tertiary follicle; FSH receptors in the oocytes, granulosa, and theca cells of tertiary follicles; FSH receptors in ovarian stroma; oxytocin receptors in oocytes and theca of secondary follicles; estrogen receptors α in the corpus luteum and tertiary follicles; estrogen receptors β in oocytes and theca of secondary follicles; estrogen receptor β in theca of tertiary follicles; estrogen receptor β in the corpus luteum; estrogen receptor β in the stroma; progesterone receptor in the oocytes, granulosa, and theca of secondary follicles; progesterone receptor in oocytes, granulosa, and theca cells of tertiary follicles; progesterone receptor in the ovarian stroma, blood progesterone, and estradiol level; and ovarian progesterone release. Therefore, flaxseed mitigated or prevented xylene’s influence on 31 (63%) indexes from a total of 49 analyzed ones. 

Flaxseed failed to substantially modify the influence of xylene on animal mortality, the diameter of primordial and primary follicles, the expression of caspase 3 in granulosa and theca cells of secondary follicles, the expression of caspase 3 in granulosa cells of tertiary follicles, FSH receptors in the oocytes of secondary follicles, estrogen receptors α in the oocytes of tertiary follicles, estrogen receptors α in the granulosa cells of tertiary follicles, estrogen receptors α in the theca of tertiary follicles, estrogen receptors α in the stroma, and ovarian IGF-I release. Therefore, flaxseed did not modify xylene’s influence on 12 (24%) indexes from a total of 49 analyzed. 

Finally, flaxseed promoted xylene’s action on the following ovarian parameters: PCNA expression in tertiary follicles, oxytocin receptors in granulosa cells of tertiary follicles, oxytocin receptors in theca cells of tertiary follicles, oxytocin receptors in the ovarian stroma, estrogen receptors α in the granulosa cells of secondary follicles, and estrogen receptors α in oocytes of tertiary follicles. Thus, flaxseed promoted xylene influence on 6 (12%) indexes from a total of 49 analyzed ones. 

These calculations demonstrate the ability of dietary flaxseed to mitigate or prevent the majority of toxic effects of xylene on ovarian functions. This plant was able to prevent the suppressive action of xylene on the growth of the ovary and its structures, as well as the ability of xylene to promote ovarian cell proliferation and their potential malignant transformation. The protective effect of flaxseed could be mediated by hormones and their receptors. This may be explained by the presence of molecules with antioxidant and phytoestrogenic properties (see above), which can prevent the action of xylene as an oxidative stressor and endocrine disrupter [2].

### 4.4. Ability of Gonadotropin to Affect Ovarian Functions and to Prevent Xylene Action 

The in vitro experiments performed during the present study confirmed the previous knowledge concerning the ability of gonadotropins to promote the release of ovarian progesterone (and the corresponding luteinization of ovarian cells) and to affect the release of IGF-I, an important regulator of ovarian functions [16,17,19]. 

Furthermore, the present studies showed the ability of gonadotropins to modify plants’ influence on ovarian hormones: the presence of gonadotropins prevented the inhibitory action of flaxseed on progesterone and estradiol and induced the inhibitory action of flaxseed on IGF-I release by murine ovaries. These observations suggest that the character of the functional food plant’s influence on reproductive processes depends on the endocrine background of an organism (the gonadotropins–ovarian hormones axis). 

The present experiments are the first demonstration of the ability of gonadotropins to prevent the suppressive influence of the environmental contaminant xylene on these hormones. The ability of gonadotropin FSH to modify the response of ovarian cells to stressors—heavy metals [30], malnutrition [31], high temperatures [31,32], and oil-related environmental contaminants [10]—has been previously reported. These observations demonstrate that gonadotropins could be another natural protector of ovarian cells against the influence of environmental stressors.

## 5. Conclusions

Taken together, the information from the present study demonstrated the influence of the environmental contaminant xylene and of functional food plant flaxseed on mortality and ovarian functions in mice. Furthermore, it shows that flaxseed could prevent the major effects of xylene on the ovary. In addition, the ability of gonadotropins to affect ovarian hormone release and to prevent its response to xylene has been shown. The effects of these additives could be mediated by changes in the release and reception of hormones. These observations suggest that flaxseed and possibly gonadotropins could be natural protectors of the female reproductive system against the adverse effects of xylene.

Nevertheless, this hypothesis requires confirmation with further experiments studying the influence of xylene and flaxseed on fecundity, functional interrelationships between various extra- and intracellular mediators of their action, and the safety and efficiency of their effects on animals and humans. It is not to be excluded that flaxseed could be useful as a natural, safe, and cheap regulator of reproductive functions and adaptogen.

## Figures and Tables

**Table 1:** Basal feed composition M3 diet and flaxseed, basic analytical and nutritive components (data of the manufacturer, BONAGRO a.s., Blažovice, Czech Republic).

M3 Diet			
Analytical Components (%)		Additional Nutritive Components in 1 kg Feed	
Crude protein	22.55	Cholecalciferol (vit. D3)	2000 IU
Crude fiber	3.14	Vitamin A	20,000 IU
Crude fat	3.31	Ferrous sulfate monohydrate	71 mg
Crude ash	5.16	Potassium iodide	0.65 mg
Ca	1.00	Coated granular cobalt bicarbonate	0.40 mg
P	0.53	Copper sulfate pentahydrate	15 mg
Na	0.11	Manganese oxide	45 mg
		Zinc oxide	71 mg
		Selenium (sodium selenite)	0.15 mg
**Flaxseed var. Libra**			
Crude protein	227.6 g/kg	Mg	4.11 g/kg
Crude fat	307.25 g/kg	Na	4.33 g/kg
Crude fiber	232.58 g/kg	K	8.12 g/kg
NDF	411.9 g/kg	P	2.71 g/kg
ADF	278.9 g/kg	Cu	33.55 mg/kg
Ash	35.5 g/kg	Zn	48.7 mg/kg
Starch	42.32 g/kg	Mn	43.83 mg/kg
Ca	2.81 g/kg	ME	12.87 MJ/kg

NDF—neutral detergent fiber; ADF—acid detergent fiber; ME—metabolizable energy.

**Table 2:** Fatty acid composition of flaxseed.

Fatty Acid	mol%
Myristic	0.05
Palmitic	4.5
Palmitoleic	0.1
Stearic	3.25
Gamma-linolenic acid	0.02
Arachidonic	0.05
Linoleic	16.65
Alfa-linolenic	57.7
Sum of n-3	72.7
Sum of n-6	8.7
n-6/n-3	0.12

**Table 3:** The effect of oral application of xylene, dietary flaxseed, and their combination on morphometric parameters (diameter in μm) of murine ovarian structures.

Ovarian Structures		Control	Xylene	Flaxseed	Xylene + Flaxseed
Whole ovary		1439 ± 104.40	1222 ± 72.22 ^a^	1133 ± 86.35 ^a^	1496 ± 54.43 ^b^
Ovarian follicles	Primordial follicle	33.97 ± 1.61	22.82 ± 3.71 ^a^	31.32 ± 1.83	19.33 ± 0.74 ^ab^
	Primary follicle	80.03 ± 2.54	67.13 ± 2.44 ^a^	68.67 ± 2.99 ^a^	69.32 ± 2.49 ^ab^
	Oocyte of primary follicle	29.40 ± 1.69	32.10 ± 1.82	30.65 ± 1.98	30.91 ± 1.87
	Secondary follicle	128.60 ± 3.92	139.20 ± 4.80 ^a^	139.20 ± 4.20 ^a^	142.70 ± 7.40 ^ab^
	Oocyte of secondary follicle	37.79 ± 2.09	43.35 ± 1.62 ^a^	35.46 ± 1.32	37.46 ± 1.86
	Tertiary follicle	223.50 ± 6.34	238.60 ± 8.80	224.40 ± 5.91	228.2 ± 6.32
	Oocyte of tertiary follicle	53.61 ± 3.04	48.64 ± 2.14 ^a^	46.96 ± 1.72 ^a^	41.73 ± 2.60 ^ab^
*Corpus luteum*		483.1 ± 43.42	395.2 ± 43.51	376.8 ± 17.63	479.3 ± 32.09

Values are mean ± SEM. ^a^—the ability of xylene or flaxseed to affect the diameter of ovarian structures: significant (*p* < 0.05) differences with control animals (without treatment); ^b^—the ability of flaxseed to modify xylene effect: significant (*p* < 0.05) differences between the animals treated with xylene alone and xylene + flaxseed.

**Table 4:** The effect of oral application of xylene, dietary flaxseed, and their combination on the expression (relative optical density, ROD) of markers of proliferation (PCNA) and apoptosis (caspase 3) in murine ovarian structures.

Analyzed Parameter	Ovarian Structure		Control	Xylene	Flaxseed	Xylene + Flaxseed
PCNA	Secondary follicle	Oocyte	1.69 ± 0.07	1.83 ± 0.07	1.92 ± 0.04 ^a^	1.63 ± 0.09 ^b^
		Granulosa	1.64 ± 0.04	1.77 ± 0.05 ^a^	1.92 ± 0.04 ^a^	1.53 ± 0.07 ^b^
		Theca	1.50 ± 0.03	1.71 ± 0.05 ^a^	1.79 ± 0.03 ^a^	1.32 ± 0.04 ^ab^
	Tertiary follicle	Oocyte	1.82 ± 0.08	1.66 ± 0.10	1.61 ± 0.05	1.65 ± 0.06
		Granulosa	1.90 ± 0.09	1.73 ± 0.06 ^a^	1.67 ± 0.06 ^a^	1.59 ± 0.05 ^ab^
		Theca	1.70 ± 0.05	1.64 ± 0.03	1.51 ± 0.07 ^a^	1.62 ± 0.03
	CL		1.53 ± 0.04	1.99 ± 0.12 ^a^	1.88 ± 0.04 ^a^	1.74 ± 0.05 ^ab^
	Stroma		1.64 ± 0.07	1.75 ± 0.04	1.74 ± 0.08	1.38 ± 0.05 ^ab^
Caspase 3	Secondary follicle	Oocyte	1.98 ± 0.10	2.35 ± 0.07 ^a^	2.12 ± 0.17	1.83 ± 0.03 ^ab^
		Granulosa	2.17 ± 0.04	2.40 ± 0.05 ^a^	2.15 ± 0.04	2.35 ± 0.11
		Theca	2.01 ± 0.05	2.26 ± 0.07 ^a^	1.94 ± 0.10	2.12 ± 0.06 ^a^
	Tertiary follicle	Oocyte	1.99 ± 0.05	1.99 ± 0.15	2.53 ± 0.07 ^a^	1.91 ± 0.03
		Granulosa	2.17 ± 0.04	2.40 ± 0.05 ^a^	2.15 ± 0.04	2.35 ± 0.11
		Theca	2.16 ± 0.02	1.95 ± 0.08 ^a^	2.33 ± 0.07 ^a^	2.21 ± 0.07 ^b^
	CL		2.26 ± 0.05	2.26 ± 0.05	2.32 ± 0.10	2.56 ± 0.03 ^ab^
	Stroma		2.11 ± 0.09	2.40 ± 0.09 ^a^	2.35 ± 0.09 ^a^	1.82 ± 0.03 ^b^

Values are mean ± SEM. CL, *corpus luteum*; ^a^—the ability of xylene or flaxseed to affect expression (ROD) of the proliferation and apoptosis marker: significant (*p* < 0.05) differences with control animals (without treatment); ^b^—the ability of flaxseed to modify xylene effect on expression (ROD) of the proliferation and apoptosis marker: significant (*p* < 0.05) differences between animals treated with xylene alone and xylene together with flaxseed.

**Table 5:** The effect of oral application of xylene, dietary flaxseed, and their combination on the expression (relative optical density, ROD) of receptors to peptide hormones (FSH receptors and oxytocin receptors) in murine ovarian structures.

Analyzed Parameter	Ovarian Structure		Control	Xylene	Flaxseed	Xylene + Flaxseed
FSH receptor	Secondary follicle	Oocyte	2.27 ± 0.04	2.87 ± 0.15 ^a^	2.62 ± 0.07 ^a^	2.69 ± 0.10 ^a^
		Granulosa	2.56 ± 0.11	2.72 ± 0.09	2.53 ± 0.04	2.63 ± 0.04
		Theca	2.53 ± 0.13	2.59 ± 0.10	2.73 ± 0.09	2.49 ± 0.02
	Tertiary follicle	Oocyte	2.05 ± 0.04	2.85 ± 0.14 ^a^	2.60 ± 0.06 ^a^	2.67 ± 0.05 ^a^
		Granulosa	2.45 ± 0.06	2.22 ± 0.06 ^a^	2.48 ± 0.06	2.68 ± 0.04 ^ab^
		Theca	2.34 ± 0.06	2.17 ± 0.06 ^a^	2.53 ± 0.07 ^a^	2.53 ± 0.06 ^ab^
	CL		2.70 ± 0.08	2.74 ± 0.08	2.59 ± 0.07	2.76 ± 0.03
	Stroma		2.34 ± 0.09	2.70 ± 0.02 ^a^	2.56 ± 0.06 ^a^	2.61 ± 0.05 ^ab^
Oxytocin receptor	Secondary follicle	Oocyte	2.28 ± 0.10	2.60 ± 0.07 ^a^	2.05 ± 0.07 ^a^	1.96 ± 0.05 ^ab^
		Granulosa	2.55 ± 0.07	2.62 ± 0.07	2.06 ± 0.05 ^a^	1.98 ± 0.03 ^ab^
		Theca	2.20 ± 0.06	2.42 ± 0.08 ^a^	1.78 ± 0.03 ^a^	1.85 ± 0.05 ^ab^
	Tertiary follicle	Oocyte	2.65 ± 0.10	2.55 ± 0.16	2.02 ± 0.02 ^a^	1.88 ± 0.04 ^ab^
		Granulosa	3.38 ± 0.12	2.67 ± 0.05 ^a^	1.81 ± 0.05 ^a^	2.03 ± 0.04 ^ab^
		Theca	2.76 ± 0.16	2.55 ± 0.04 ^a^	1.79 ± 0.03 ^a^	1.87 ± 0.03 ^ab^
	CL		3.43 ± 0.17	2.35 ± 0.08 ^a^	2.01 ± 0.04 ^a^	2.03 ± 0.03 ^ab^
	Stroma		2.72 ± 0.13	2.49 ± 0.05 ^a^	1.84 ± 0.02 ^a^	1.86 ± 0.06 ^ab^

Values are mean ± SEM. CL, *corpus luteum*; ^a^—the ability of xylene or flaxseed to affect expression (ROD) of receptors: significant (*p* < 0.05) differences with control animals (without treatment); ^b^—the ability of flaxseed to modify the xylene effect on expression (ROD) of receptors: significant (*p* < 0.05) differences between animals treated with xylene alone and xylene together with flaxseed.

**Table 6:** The effect of oral application of xylene, dietary flaxseed, and their combination on the expression (relative optical density, ROD) of estrogen receptors (ER) alpha, beta, and progesterone receptor (PR) B in murine ovarian structures.

Analyzed Parameter	Ovarian Structure		Control	Xylene	Flaxseed	Xylene + Flaxseed
ERα	Secondary follicle	Oocyte	1.44 ± 0.03	1.53 ± 0.08	1.52 ± 0.04	1.58 ± 0.01
		Granulosa	1.64 ± 0.09	1.52 ± 0.02 ^a^	1.64 ± 0.03	1.49 ± 0.03 ^ab^
		Theca	1.57 ± 0.07	1.62 ± 0.06	1.73 ± 0.04	1.56 ± 0.04
	Tertiary follicle	Oocyte	1.27 ± 0.04	1.58 ± 0.04 ^a^	1.49 ± 0.05 ^a^	1.52 ± 0.06 ^a^
		Granulosa	1.48 ± 0.03	1.56 ± 0.03 ^a^	1.56 ± 0.04 ^a^	1.60 ± 0.02 ^a^
		Theca	1.43 ± 0.04	1.64 ± 0.05 ^a^	1.64 ± 0.05 ^a^	1.65 ± 0.06 ^a^
	CL		1.61 ± 0.03	1.92 ± 0.04 ^a^	1.68 ± 0.03 ^a^	1.75 ± 0.02 ^ab^
	Stroma		1.45 ± 0.04	1.62 ± 0.05 ^a^	1.63 ± 0.05 ^a^	1.69 ± 0.04 ^a^
ERβ	Secondary follicle	Oocyte	1.51 ± 0.04	2.59 ± 0.05 ^a^	2.53 ± 0.17 ^a^	2.18 ± 0.06 ^ab^
		Granulosa	1.53 ± 0.05	2.18 ± 0.10 ^a^	2.02 ± 0.05 ^a^	2.13 ± 0.07 ^a^
		Theca	1.50 ± 0.04	2.15 ± 0.03 ^a^	2.11 ± 0.07 ^a^	2.04 ± 0.06 ^ab^
	Tertiary follicle	Oocyte	1.44 ± 0.06	1.76 ± 0.05 ^a^	2.32 ± 0.10 ^a^	1.98 ± 0.06 ^ab^
		Granulosa	1.51 ± 0.05	2.15 ± 0.05 ^a^	2.12 ± 0.07 ^a^	2.07 ± 0.03 ^a^
		Theca	1.40 ± 0.02	2.30 ± 0.03 ^a^	2.30 ± 0.09 ^a^	2.04 ± 0.08 ^ab^
	CL		1.47 ± 0.02	2.71 ± 0.05 ^a^	2.27 ± 0.04 ^a^	2.31 ± 0.07 ^ab^
	Stroma		1.67 ± 0.11	2.24 ± 0.07 ^a^	2.35 ± 0.05 ^a^	2.00 ± 0.07 ^ab^
PRB	Secondary follicle	Oocyte	1.79 ± 0.04	2.36 ± 0.10 ^a^	1.75 ± 0.11	1.82 ± 0.07
		Granulosa	1.94 ± 0.06	2.68 ± 0.09 ^a^	1.62 ± 0.03 ^a^	1.82 ± 0.04 ^ab^
		Theca	1.78 ± 0.06	2.64 ± 0.07 ^a^	1.63 ± 0.02 ^a^	1.68 ± 0.03 ^ab^
	Tertiary follicle	Oocyte	1.97 ± 0.12	2.25 ± 0.08 ^a^	1.94 ± 0.03	2.15 ± 0.02
		Granulosa	2.16 ± 0.06	2.97 ± 0.09 ^a^	1.74 ± 0.03 ^a^	1.92 ± 0.02 ^ab^
		Theca	2.08 ± 0.07	2.73 ± 0.05 ^a^	1.65 ± 0.04 ^a^	1.81 ± 0.03 ^ab^
	CL		2.19 ± 0.06	2.26 ± 0.05	1.60 ± 0.04 ^a^	1.75 ± 0.02 ^ab^
	Stroma		1.55 ± 0.05	2.57 ± 0.07 ^a^	1.56 ± 0.07	1.78 ± 0.04 ^ab^

Values are mean ± SEM. CL, *corpus luteum*; ^a^—the ability of xylene or flaxseed to affect expression (ROD) of receptors: significant (*p* < 0.05) differences with control animals (without treatment); ^b^—the ability of flaxseed to modify xylene effect on expression (ROD) of receptors: significant (*p* < 0.05) differences between animals treated with xylene alone and xylene together with flaxseed.

## Data Availability

All data related to this study are presented in the main text.
